# Pharmacists’ Knowledge and Perceptions of FDA Approval Standards and the Breakthrough Therapy Designation

**DOI:** 10.3390/pharmacy10050126

**Published:** 2022-09-30

**Authors:** Megan C. Herink, Kirbee Johnston, Kristin Breninger, Erin Wu, Adriane N. Irwin

**Affiliations:** Department of Pharmacy Practice, Oregon State University, 1601 SW Jefferson Way, Corvallis, OR 97331, USA

**Keywords:** breakthrough therapy designation, drug approval, drug labeling, pharmacists, United States Food and Drug Administration

## Abstract

The “breakthrough therapy” designation (BTD) is a recent mechanism implemented by the United States Food and Drug Administration (FDA) to expedite access to drugs that address unmet needs. The purpose of this study is to describe pharmacists’ knowledge of FDA drug-approval standards and knowledge and perceptions of the BTD. Pharmacists engaged in advanced clinical practice were identified through membership profiles of a professional pharmacy organization. Eligible participants were then sent a questionnaire to assess knowledge of FDA approval standards and the BTD. A total of 226 pharmacists responded. The majority of respondents were women (70.2%) and had completed post-graduate training (85.8%). Over half correctly answered at least two of three questions on FDA approval standards (58.1%) and the BTD (78.1%). Only 24.1% of respondents identified as being familiar with the BTD. The majority of pharmacists (62.8%) were certain that FDA-approved “breakthrough” drugs represented a major advance over currently approved therapies and most (88.5%) preferred the drug designated as “breakthrough” in a hypothetical scenario. In conclusion, pharmacists were able to correctly answer questions about FDA approval standards and the BTD. However, they were unfamiliar with the implications of a BTD and may overestimate the benefit demonstrated by these drugs. Future research should identify knowledge gaps in pharmacist understanding of regulatory mechanisms designed to expedite drug approval.

## 1. Introduction

New drugs must be proven safe and efficacious by the Food and Drug Administration (FDA) prior to market release and widespread use by patients in the United States (US) [1]. This process assists healthcare professionals, patients, and other stakeholders in making informed decisions by outlining the anticipated clinical benefits and possible risks of a medication [1,2,3]. One criticism of the process is the length of time often required for drugs to obtain FDA approval, and the resulting delay in access to therapies. In response, Congress has modified the Federal Food, Drug, and Cosmetic Act to expedite development and approval of therapies that address unmet needs [4,5]. Created in 2012 through the FDA Safety and Innovation Act, the “breakthrough therapy” designation (BTD) is a recent addition to these approaches [5,6].

Eligibility for a BTD requires that a drug have preliminary clinical evidence which may demonstrate a substantial improvement over current therapies based on a surrogate endpoint(s), pharmacodynamic biomarker(s), or a significantly improved safety profile [4,5]. This designation provides advantages through additional guidance and support from FDA officials in designing clinical trials, as well as benefits from other mechanisms designed to reduced time to FDA approval if criteria are met, including fast-track designation [4,5]. These approval features include early FDA guidance on efficient drug development, organizational commitment from senior agency officials, and eligibility for priority and rolling review [4]. To support BTD features, the FDA may meet frequently with the drug sponsor throughout drug development and involve experienced staff in a cross-disciplinary review to maintain a short development program [4]. With 225 BTD drugs approved as of December 31, 2021, the BTD has been successful in facilitating approval of therapies for conditions with limited treatment options, which are often also rare and/or serious [6,7,8].

Concerns relating to the BTD, such as the limited safety and efficacy data required for approval and high costs of these medications, have been raised [8,9,10,11]. More recently, it has been highlighted that the required post-marketing confirmatory studies have frequently been delayed or not completed at all [12]. Additionally, inconsistencies in regulatory consequences for drugs not proving beneficial in follow-up studies has resulted in unproven indications in the drug label [13]. Until confirmatory trials demonstrate safety and efficacy, health care providers should be aware of the level of uncertainty with drugs approved with a BTD to accurately communicate anticipated benefits with patients and other providers.

Prescriber knowledge of the evidence behind a medication’s approval is vital for appropriate patient care, and research has suggested that use of the term “breakthrough” can lead to an overestimation of the known efficacy of a drug by physicians and consumers [14,15,16]. Healthcare is becoming more interprofessional, and pharmacists are increasingly positioned to contribute to the development of treatment plans by weighing safety and efficacy data. The American Society of Health-System Pharmacists (ASHP) states that the provision of drug information is a fundamental responsibility of all pharmacists and includes establishing and maintaining a formulary based on scientific evidence as part of optimal drug information services [17]. Pharmacists are routinely asked by healthcare professionals about the benefits and risks of new drugs that come to market, which are more commonly targeting rare diseases [18]. As a result, it is important to understand pharmacists’ knowledge of the meaning of FDA approval and the BTD designation. The purpose of this study is to describe pharmacists’ knowledge of FDA approval standards and knowledge and perceptions of the BTD.

## 2. Materials and Methods

### 2.1. Study Design and Participants

This was a cross-sectional survey of pharmacists who identified as engaged in clinical pharmacy practice in the US. The survey distribution list was created by manually reviewing membership profiles (available to all members of the organization) within a professional pharmacy organization focused on clinical pharmacy practice. Profiles of individuals who self-identified as practicing in ambulatory care, hematology and oncology, and adult internal medicine were selected. These clinical areas were identified as highly impacted by BTD drug approvals. If the profile contained an indicator that the member was a pharmacist via degree (e.g., PharmD), license (e.g., RPh), or credential (e.g., board certification), then the member’s email address was extracted (if available) to develop the distribution list. Profiles with a location outside of the US were excluded.

Identified pharmacists were sent an email in March 2019 containing a short description of the study and a link to an online survey (Qualtrics, Provo, UT). Two additional emails were sent at one to two-week intervals to non-responders to encourage completion of the survey. Upon survey conclusion, respondents could elect to be entered into a drawing for one of four USD 100 gift cards to provide incentive for survey participation.

### 2.2. Survey Development and Analysis

The survey instrument consisted of questions in three areas: (1) demographic characteristics, (2) knowledge of FDA approval standards and the BTD, and (3) response to a hypothetical scenario of a newly approved drug to evaluate the impact of using the “breakthrough” term. The hypothetical scenario did not have a correct answer. Respondents were asked to choose between a drug described as “breakthrough” or a drug described using the detailed definition of BTD. Except for the demographic questions, the survey was duplicated, with permission from the corresponding author, from an existing survey designed to assess physician knowledge of FDA approval standards and perceptions of the BTD [14]. The instrument consisted of 15 items and took 5–10 min to complete. Respondents were included in the analysis if they answered two or more questions.

All data were collected from the online survey instrument. Categorical data are summarized with descriptive statistics. Data analysis was performed using SAS statistical software (version 9.4; SAS Institute) All study activities were reviewed and approved by the Oregon State University Institutional Review Board.

## 3. Results

A total of 232 of 2361 pharmacists responded to the survey. Of those, six respondents did not answer more than one question and were excluded from the analysis. This left a total of 226 respondents for a response rate of 9.6%. Respondent demographics are presented in Table 1. The majority of respondents were female (*n* = 158 of 225; 70.2%), post-graduate trained (*n* = 193 of 225; 85.8%), and board certified (*n* = 188 of 223; 84.3%). The greatest number of respondents had been in practice for less than 5 years (*n* = 79 of 224; 35.3%); however, there was a range of experience with 51 (22.8%) respondents having 5–10 years, 20 (8.9%) respondents having 10–15 years, and 74 (33.0%) respondents having more than 15 years of practice experience. Few respondents (*n* = 22 of 225; 9.8%) identified as a current trainee in a post-graduate program. There was also representation from the various geographic regions of the US.

Pharmacist knowledge of FDA approval standards is summarized in Table 2. The majority of respondents were aware that FDA approval typically means that a drug’s benefits outweigh the harms (*n* = 193 of 212; 91.0%), and it does not mean that a drug is proven as effective as other approved drugs (*n* = 123 of 212; 58.0%). However, respondents did frequently incorrectly indicate that FDA approval requires results to be clinically significant, statistically significant, or both (*n* = 176 of 212; 83.0%). Overall, pharmacists demonstrated reasonable knowledge of FDA approval standards, with over half (*n* = 123 of 212; 58.0%) answering at least two of the three questions correctly.

Pharmacist knowledge of the BTD is summarized in Table 3. Most respondents were not familiar with the BTD (*n* = 160 of 211; 75.8%) and responded as either “a little familiar” or “not familiar at all.” However, despite this self-reported unfamiliarity, the majority of respondents (*n* = 164 of 210; 78.1%) correctly answered at least two of three questions about the quality of evidence required for a BTD. Respondents correctly identified that a BTD only requires preliminary evidence (*n* = 141 of 211; 66.8%), and it does not require evidence that the drug is neither more effective (*n* = 141 of 210; 67.1%) nor safer than alternatives (*n* = 183 of 211; 86.7%). While there is no certainty that a BTD approval will result in a major medical advancement, most respondents (*n* = 133 of 212; 62.7%) reported being “fairly certain” or “very certain” of otherwise. Finally, in the hypothetical scenario, the majority of respondents selected the option that used the “breakthrough” term (*n* = 185 of 209; 88.5%), rather than the description of the BTD that communicated the limited proven benefit at the time of the approval.

## 4. Discussion

It is important to have regulatory mechanisms that facilitate timely development and approval of drugs that address unmet needs, particularly drugs for serious and life-threatening conditions with inadequate therapies. However, it is also important to patients and providers alike to have a safe and effective FDA approval process. Despite an increasing use of mechanisms designed to shorten time to FDA approval, there is no research into pharmacist knowledge or understanding of these different mechanisms nor how these mechanisms differ in requirements for data on clinically meaningful endpoints. This national survey demonstrates that pharmacists understand FDA approval standards, and despite unfamiliarity with the BTD, some awareness of variations in evidentiary requirements for drug approval. However, responses also suggest a potential overestimation of the proven benefits of BTD drugs and impact of describing a medication as “breakthrough” on health professionals’ attitudes.

While a lack of similar research makes synthesis of these results with existing literature challenging, some interesting comparisons do exist. Questions were taken from a similar cross-sectional survey conducted on physicians [14]. In that work, only 27% and 46% of physician respondents answered two or more questions correctly about FDA approval standards and the BTD, respectively, as compared to 58% and 78% of pharmacists in this study [14]. The improved performance of pharmacists on these questions is not surprising given a greater focus on drugs, including regulatory requirements, in pharmacy education, as well as the role pharmacists play as drug information experts on interprofessional care teams [17,19]. However, it was notable that 62.7% of pharmacists incorrectly felt “very certain” or “fairly certain” that a “breakthrough” drug represents a major advance over other approved treatments. Similarly, 88.5% of respondents selected the option that used the term “breakthrough,” rather than the description of the term, in the hypothetical scenario, which has been associated with an increased belief in a drug’s effectiveness in other research [14,15]. As a result, while pharmacists may have broad knowledge of drug-approval standards, they may be lacking the more nuanced understanding that is required to fully weigh efficacy and safety of a drug approved through an expedited process.

A gap in pharmacist understanding of regulatory information would be consistent with other literature that has demonstrated knowledge gaps related to the FDA’s Pregnancy and Lactation Labeling Rule changes, therapeutic equivalence standards for generic medications, and adverse drug event (ADE) reporting through the FDA’s MedWatch program, as well as limited acceptance of biosimilar medicines [20,21,22,23]. Pharmacists are assuming larger roles in the development of treatment plans and interacting with patients and providers, so understanding the nuances of how drugs are FDA approved is increasingly important. Specific to this work, while the term “breakthrough” is generally defined as an important discovery or development, this definition is different than the FDA’s usage because FDA-designated “breakthrough” drugs have only demonstrated a potential to provide substantial improvement over existing therapies. Concern about the use of the term “breakthrough” was first raised in research with consumers using different versions of an FDA press release for a metastatic cancer drug. Consumers randomized to press releases with the term “breakthrough” or “promising” were more likely to believe in a drug’s effectiveness and strength of supporting evidence, a phenomenon that has been further replicated in a survey of physicians [14,15]. As a result, while the hypothetical scenario involving Axabex and Zykanta presents a false dilemma, as one option uses the term “breakthrough” while the other relies upon the FDA’s definition, the strong preference toward Axabex, rather than equal option selection, suggests that the term “breakthrough” may have similar influence among pharmacists.

Research into pharmacist knowledge on regulatory topics is unfortunately sparse and often limited in scope. However, the knowledge gaps that have been identified can translate to behavior that negatively impacts patients. Pharmacovigilance and ADE reporting is essential to fully characterize the safety profile of drugs; as a result, the lack of serious ADE reporting by pharmacists could delay identification of medication safety concerns [22]. Similarly, the reluctance to embrace biosimilars due to a lack of comfort with these products can result in delays to effective therapy options and/or higher costs to patients [23]. Regulatory affairs, including federal statutes and regulations that regulate the practice of pharmacy, are central to contemporary pharmacy education and required by current Accreditation Council for Pharmacy Education standards (also known as, Standards 2016) [19]. However, unlike other areas essential to pharmacy practice, such as education on substance use disorders, there has been no systematic evaluation of how education on FDA approval standards and other complementary regulatory topics is delivered within schools and colleges of pharmacy nor how this content has evolved over time [24]. Educational initiatives to support both practicing pharmacists, as well as pharmacy learners, on FDA approval pathways are likely needed to help pharmacists best participate in advanced practice roles. Research into what content on FDA approval standards is currently taught and the depth would be an opportunity for future research.

Finally, highlighted by the COVID-19 pandemic, pharmacists are frequently on the frontline for interacting and communicating information to the public and other healthcare providers. Pharmacists were vital in providing accurate and reliable information about off-label and supportive treatments for COVID-19, including roles in dispelling misinformation about hydroxychloroquine and ivermectin [25]. They have also needed to understand regulatory tools, specifically the FDA’s use of emergency use authorizations (EUA), designed to make access to drugs and vaccines faster [26]. While the FDA’s decision to allow early access to COVID-19 vaccines and other treatments unquestionably saved lives, similar to drugs approved with a BTD, not all EUA-approved agents have been without controversy. Even before bamlanivimab’s EUA was revoked due to ineffectiveness against variants, concerns were raised about its questionable efficacy and incomplete safety profile [27].

This study is not without limitations, including survey response biases. The response rate was low which increases the risk of non-response bias, although it should be noted that respondents were from a variety of geographical locations and had different levels of experience. The response rate is also greater than other surveys that have used membership in a specific professional pharmacy organization to develop a representative sample. For example, a recent survey on buprenorphine dispensing practices by community pharmacists that used membership in the American Pharmacists Association, only had response rate of 5.1% [28]. Similarly, although the majority of participants were female and had been practicing less than 10 years, this is consistent with trends in the pharmacy profession in the US. Pharmacy is a female dominated profession and has experienced rapid growth in conferring the entry-level professional degree (i.e., Doctor of Pharmacy) over the last few decades [29,30]. Respondents were also members of a single professional organization and had self-identified as practicing in specific areas. This was intentional, as the authors were attempting to identify pharmacists with the greatest likelihood of engaging with a BTD-approved drugs outside of the traditional dispensing process. However, the knowledge of those in this sample may not be reflective of pharmacists practicing in other settings. Lastly, it is an imperfect comparison between the prescriber survey and pharmacist survey due to differences in clinical specialties, survey response rates, and time since inception of BTD. Since the initial prescriber survey was completed over five years ago, providers and healthcare professionals could have gained more familiarity with the BTD. However, a more recent follow up survey found that among mostly primary care physicians, approximately one third still incorrectly assumed a higher level of evidence behind the BTD and there remained a disproportionate response favoring the new drug described as “breakthrough” [16].

## 5. Conclusions

In this nationwide survey of pharmacists practicing in specific clinical settings, pharmacists were able to correctly answer questions about FDA approval standards and the BTD. However, many pharmacists were unfamiliar with the implications of the BTD and may overestimate the benefit demonstrated by these drugs. Future research should identify knowledge gaps in pharmacist understanding of FDA pathways, specifically those designed to increase access to therapies for rare and/or serious conditions with limited treatment options, so that pharmacists are optimally positioned to fully assess the safety and efficacy of these drugs when developing treatment plans and communicating with patients and other healthcare professionals. Furthermore, additional research evaluating knowledge gaps and educational needs in other practice settings and educational environments is needed to better understand barriers and opportunities.

## Figures and Tables

**Table 1:** Respondent Demographics, *n* (%).

Characteristic	Response
Gender (*n* = 225)	
Female	158 (70.2)
Male	64 (28.4)
Prefer not to answer	3 (1.3)
Current post-graduate trainee, ^1^ yes (*n* = 225)	22 (9.8)
Completed a post-graduate program, ^1^ yes (*n* = 225)	193 (85.8)
Board certified, yes (*n* = 223)	188 (84.3)
Years in practice, excluding post-graduate training (*n* = 224)	
Less than 5 years	79 (35.3)
5–10 years	51 (22.8)
10–15 years	20 (8.9)
More than 10 years	74 (33.0)
Region of the United States (*n* = 225)	
Northeast	40 (17.8)
Southeast	50 (22.2)
Midwest	67 (29.8)
Northwest	22 (9.8)
Southwest	44 (19.6)
Other	2 (0.9)

^1^ Post-graduate training defined in the question as a residency, fellowship, or other advanced degree.

**Table 2:** Pharmacist knowledge of United States Food and Drug Administration’s (US FDA) approval standards, *n* (%).

US FDA Approval Survey Questions	Response
FDA approval generally means that a drug is as effective as other drugs approved to treat the same condition. (*n* = 212)	
True	89 (42.0)
False *	123 (58.0)
FDA approval generally means that a drug has benefits that outweigh its harms. (*n* = 212)	
True *	193 (91.0)
False	19 (9.0)
In order for a drug to be FDA approved, it has to have… (*n* = 212)	
A statistically significant result.	50 (23.6)
A clinically significant result.	41 (19.3)
Both of the above	85 (40.1)
None of the above *	36 (17.0)
Pharmacists with number of correct answers (*n* = 212)	
0 questions	2 (0.9)
1 question	87 (41.0)
2 questions	104 (49.1)
3 questions	19 (9.0)

* Indicates the correct answer.

**Table 3:** Pharmacist knowledge and perceptions of the breakthrough therapy designation, *n* (%).

Breakthrough Therapy Designation Survey Questions.	Response
Prior to taking this survey, how familiar were you with the “breakthrough drug” designation? (*n* = 211)	
Very familiar	10 (4.7)
Familiar	41 (19.4)
A little familiar	87 (41.2)
Not familiar at all	73 (34.6)
In general, I am certain that an FDA-approved “breakthrough drug” represents a major advance over currently approved treatments for the same indication. (*n* = 212)	
Very certain	22 (10.4)
Fairly certain	111 (52.4)
Fairly uncertain	60 (28.3)
Very uncertain	19 (9.0)
What is the minimum level of evidence that the FDA requires manufacturers to gather in order for the FDA to label a drug as a “breakthrough?” (*n* = 211)	
Strong evidence (e.g., randomized trials evaluating clinical outcomes)	53 (25.1)
Preliminary (e.g., uncontrolled studies or studies testing surrogate outcomes) *	141 (66.8)
Very preliminary (e.g., animal studies)	17 (8.1)
When the FDA calls a drug a “breakthrough,” does that mean that there is high quality evidence that the drug is more effective than currently approved treatments? (*n* = 210)	
True	69 (32.9)
False *	141 (67.1)
When the FDA calls a drug a “breakthrough,” does that mean that there is high quality evidence that the drug is safer than currently approved treatments? (*n* = 211)	
True	28 (13.3)
False *	183 (86.7)
Pharmacists with number of correct answers (*n* = 210)	
0 questions	13 (6.2)
1 question	33 (15.7)
2 questions	62 (29.5)
3 questions	102 (48.6)
Hypothetical Scenario: Imagine your patient has a serious medical condition for which there has been no effective treatment. The FDA recently approved 2 new drugs to treat this condition. Both drugs are oral tablets to be taken once daily, have similar side effect profiles, and are equally covered by the patient’s insurance. Which would you choose first? (*n* = 209)	
Axabex, an FDA-designated “breakthrough” drug	185 (88.5)
Zykanta, a drug with early promising study results but which has not been shown to improve survival or disease- related symptoms	24 (11.5)

* Indicates the correct answer.

## Data Availability

Not applicable.
